# Missed Opportunities: Poor Linkage into Ongoing Care for HIV-Positive Pregnant Women in Mwanza, Tanzania

**DOI:** 10.1371/journal.pone.0040091

**Published:** 2012-07-09

**Authors:** Deborah Watson-Jones, Rebecca Balira, David A. Ross, Helen A. Weiss, David Mabey

**Affiliations:** 1 Department of Clinical Research, London School of Hygiene and Tropical Medicine, London, United Kingdom; 2 Mwanza Intervention Trials Unit, National Institute for Medical Research, Mwanza, Tanzania; 3 Department of Sexual and Reproductive Health, National Institute for Medical Research, Mwanza, Tanzania; 4 MRC Tropical Epidemiology Group, Department of Infectious Disease Epidemiology, London School of Hygiene and Tropical Medicine, London, United Kingdom; Vanderbilt University, United States of America

## Abstract

**Background:**

Global coverage of prevention of mother-to-child (PMTCT) services reached 53% in 2009. However the number of pregnant women who test positive for HIV in antenatal clinics and who link into long-term HIV care is not known in many resource-poor countries. We measured the proportion of HIV-positive pregnant women in Mwanza city, Tanzania, who completed the cascade of care from antenatal HIV diagnosis to assessment and engagement in care in adult HIV clinics.

**Methods:**

Thirty antenatal and maternity ward health workers were interviewed about PMTCT activities. Nine antenatal HIV education sessions were observed. A prospective cohort of 403 HIV-positive women was enrolled by specially-trained clinicians and nurses on admission to delivery and followed for four months post-partum. Information was collected on referral and attendance at adult HIV clinics, eligibility for highly active antiretroviral therapy (HAART) and reasons for lack of attendance.

**Results:**

Overall, 70% of PMTCT health workers referred HIV-positive pregnant women to the HIV clinic for assessment and care. Antenatal HIV education sessions did not cover on-going care for HIV-infected women. Of 310 cohort participants tested in pregnancy, 51% had received an HIV clinic referral pre-delivery. Only 32% of 244 women followed to four months post-partum had attended an HIV clinic and been assessed for HAART eligibility. Non-attendance for HIV care was independently associated with fewer antenatal visits, poor PMTCT prophylaxis compliance, non-disclosure of HIV status, and non-Sukuma ethnicity.

**Conclusion:**

Most women identified as HIV-positive during pregnancy were not assessed for HAART eligibility during pregnancy or in the first four months post-partum. Initiating HAART at the antenatal clinic, improved counselling and linkages to care between PMTCT and adult HIV treatment services and reducing stigma surrounding disclosure of HIV results would benefit on-going care of HIV-positive pregnant women.

## Introduction

**Figure 1:**
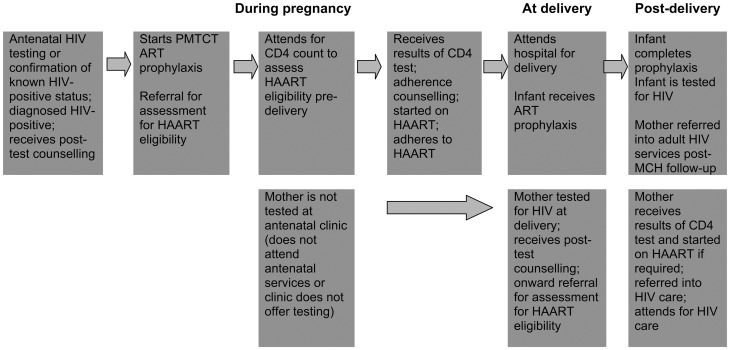
Cascade of referral and care for HIV-positive pregnant women in Tanzan.

Mother-to-child transmission (MTCT) of HIV has been virtually eliminated in developed countries through effective prevention of mother-to-child transmission (PMTCT) programmes, but, although countries such as Botswana have demonstrated considerable success in reducing MTCT rates, significant challenges and gaps in service remain in most developing countries [1]–[6]. The 2010 World Health Organisation PMTCT guidelines recommend that all HIV-positive pregnant women should be assessed for eligibility to start highly active antiretroviral therapy (HAART) for their own health via referral to an HIV care and treatment centre [7]. PMTCT programmes therefore represent a major opportunity to go beyond the prevention of infant infections to allow diagnosis and management of previously-unrecognised maternal HIV infection, and the prevention of HIV-related orphans. However, this requires that women navigate a series of steps along the cascade from testing, diagnosis, assessment for eligibility for life-long HAART, to initiation of HAART if indicated (Figure 1).

Data from South Africa on the operational performance of onward referral from PMTCT services of women identified as HIV-positive in pregnancy suggest that, whilst a high proportion of pregnant women may be HIV tested and receive ART prophylaxis for PMTCT at the ANC, a significant proportion are not assessed during pregnancy for eligibility to receive lifelong HAART or do not receive HAART if they are assessed as needing it [8], [9]. Linkage to HIV care and treatment following testing at non-antenatal clinic settings in sub-Saharan Africa has also been a challenge, including after general provider-initiated testing and counselling, testing of TB patients and through voluntary counselling and testing services [10]–[14]. As part of a study examining PMTCT services within public health facilities in northwest Tanzania, we aimed to measure whether HIV-positive women identified through PMTCT received appropriate referral and care for their own health concerning HIV, and to explore individual and provider barriers that could explain why they did not attend.

## Methods

The study was located in Mwanza city in Tanzania, a low-income country with an estimated adult HIV prevalence of 5.6% [15], [16]. Mwanza, Tanzania’s second largest city, had a population of 474,797 at the last census in 2002 [17] and has an estimated HIV prevalence in pregnant women of 8.4% [18]. Overall, 98% of Tanzanian pregnant women are reported to attend an antenatal clinic at least once in pregnancy, approximately 43% to make four or more visits and 82% of urban women deliver in hospital [19].

Data were collected between September 2008 to July 2009 from health worker interviews, from observation of PMTCT antenatal services at three antenatal clinics in Mwanza and from a cohort study of HIV-positive women recruited at the maternity wards of two large government hospitals in Mwanza city. These two hospitals also housed the main adult HIV care and treatment clinics for the city. All three antenatal clinics had an adult HIV clinic on site. In two of these sites the HIV clinics had recently opened. However, because of concern that patients might not like to attend a clinic near their place of residence, pregnant women were usually asked where they would like to attend and could be therefore be referred to HIV clinic services 5–10 km away.

### Ethical Considerations

The study was approved by the Ethics Committees of the Tanzanian National Institute for Medical Research and the London School of Hygiene & Tropical Medicine. Informed written or finger-printed consent was obtained from all cohort participants.

### Health Worker Interviews

A structured questionnaire with open and closed questions was administered to all health workers at the three largest antenatal clinics and the maternity wards of the two largest government hospitals in Mwanza city by RB or a trained nurse or clinician interviewer. Data gathered included key work activities, PMTCT and other training received, perceived challenges in implementing PMTCT services and PMTCT-related knowledge.

### Cohort Study

We enrolled 403 of 442 HIV positive women admitted for delivery between September 2008 and February 2009.

HIV status was routinely recorded in the antenatal card that pregnant women brought with them on admission for delivery and these results were routinely recorded in the maternity ward admissions book. All consecutively-admitted eligible HIV-positive women were seen in private by a counsellor to inform them about the study. Women were not eligible if they were resident outside Mwanza city or were unable to provide informed consent. Of the 39 women not enrolled, 27 lived outside Mwanza, 6 refused, 4 were missed, and 2 were unable to give informed consent.

Those who agreed to participate were given further verbal and written information about the study by a research assistant and were asked to sign or fingerprint a consent form. They were interviewed about socio-demographic characteristics, outcomes of previous pregnancies and past contraceptive use. Information collected about the current pregnancy included details on antenatal care, uptake of PMTCT services, referral and attendance at an adult HIV clinic during that pregnancy, CD4 cell count results if available and HAART status. In the currently-used 2007 Tanzanian National PMTCT guidelines, eligibility for HAART is based on a CD4 cell count of less than 200 cells/mm^3^ or being diagnosed as having WHO Stage 3 or 4 [20]. Women who are not eligible for HAART should be offered zidovudine (AZT) from 28 weeks gestation, single dose (sd) nevarapine (NVP) and 3TC at labor onset and through delivery and continued for one week post-partum, with the infant receiving sdNVP after delivery and AZT syrup for 4 weeks (or one week if mother had received 4 or more weeks of AZT). At the time of the study, provider-initiated counselling and testing had been introduced with all women being referred into a PMTCT counsellor at their first antenatal visit in that pregnancy.

Prior to discharge from hospital, data were collected on whether the mother had been tested for HIV in the maternity suite and on any onward referral. Enrolled women were followed up at their monthly visits at the under-five reproductive and child health (RCH) clinic or four months post-partum. At each visit, participants were interviewed about referral, disclosure of HIV status to others, their progress at the HIV clinic and were asked to show the interviewer their HIV clinic card which documented attendance dates, CD4 cell count results by date, and date of initiation of HAART, if applicable. Those who had not attended an HIV clinic were asked about reasons for lack of attendance. They were then re-referred to the counsellor at the RCH clinics to reiterate why assessment of their own health was important and to encourage them to attend the HIV clinic for this. They were followed up at home by a field worker if they did not attend the clinic visit and had given consent for home visits. If the field worker believed that the interview could not be conducted in private at the home then the interview was postponed and the participant was asked to come to the health facility.

The study aimed to enroll 330 HIV-positive women. Allowing for a 20% loss to follow-up over four months, it was estimated that 264 women attending the four month visit would allow us to measure the proportion of HIV-positive pregnant women referred to and attending an HIV clinic with adequate precision if the proportion attending HIV clinic services was between 40–70%.

### Data Management

Data were double-entered in EpiData 3.1 (Epidata Association, Odense, Denmark) and cleaned and analysed using Stata 11.0 (STATA Corporation, Texas, USA). Data from the cohort study were used to determine the proportion of women who successfully completed the PMTCT/maternal health uptake intervention cascade. A Piot-Fransen model was built up to show the fall-off at each step of the referral and assessment cascade. This used the denominator of women who successfully completed the previous step based on data from women who tested HIV-positive for the first time during the current pregnancy and who were seen at the fourth monthly post-partum follow-up visit. This was to allow inclusion of women who had complete information up to the end of the study. This model was adapted from an evaluation model of case finding and treatment for tuberculosis control programmes and has been used to evaluate other programmes such as those for sexually transmitted diseases [21], [22].

To identify potential client and provider factors in the cohort study that were associated with successful completion of the referral and treatment cascade, crude odds ratios (OR) with 95% CI were estimated using logistic regression analysis. Factors whose univariable association with the outcome reached statistical significance at p<0.1 were included in an initial multivariate model and retained in the final model if independently associated with the outcome (p<0.1). Associations with p-values below 0.01 are interpreted as showing strong evidence, p-values around 0.05 as moderate evidence, and p-values around 0.1 as weak evidence.

## Results

### Cohort Study: HIV Clinic Referral and Attendance

In total, 403 HIV-positive women admitted for delivery were enrolled. The mean age of cohort participants was 28.3 years (range 16 to 48 years). Seven women did not know their age. Overall, 164 (40.7%) were Catholic, 140 (34.7%) were other Christians, 97 (24.1%) were Muslims and 2 (0.5%) had no religion. Most (n = 365; 90.6%) had primary school or higher education; 192 (47.6%) were housewives, 196 (48.6%) reported having unskilled work and 15 (3.7%) reported being in skilled work. Two hundred and eighty five (70.7%) were married, 47 (11.7%) were single, 53 (13.2%) were divorced or separated and 18 (4.5%) were widowed. Two hundred and twenty seven (56.3%) were Sukuma, the largest ethnic group in Mwanza Region.

Overall 93 of 403 enrolled women reported that they had been tested and diagnosed with HIV before the current pregnancy and were excluded from this analysis. Of the remaining 310 women who tested HIV-positive through PMTCT services, 240 (77.4%) were tested during pregnancy, 23 (7.4%) were tested at the maternity ward before delivery, and 47 (15.2%) were tested at the maternity ward after delivery.

At enrollment, of the 240 women who were tested at antenatal PMTCT services, 123 (51.3%) had received a referral to an HIV clinic. Of those referred, 71 (57.7%) had attended prior to delivery of whom only 36 (50.7%) could confirm their attendance by providing a clinic card. Only 30 (24.3%) of those referred (83.3% of those with a clinic card) had evidence of a CD4 test result (Table 1). Five (16.7%) women had a CD4 cell count result of less than 200 cells/mm^3^, three of whom (60.0%) had been started on HAART.

In total, 70/310 previously-untested HIV-positive women were tested at the maternity ward (22.6%). No referrals to the HIV clinic were given by maternity ward staff to HIV-positive women who had not yet attended HIV care and treatment services, including those who were identified as HIV-positive for the first time in the maternity ward.

A total of 244 (79.0%) women were seen at the four month follow-up visit, and by this time 199 (81.6%) had received referral to an HIV clinic. Approximately half received this referral pre-delivery and half received the referral post-delivery, when they attended the under-five clinic or when they visited the maternity wards for infant diagnosis (Table 1).

Overall 133 (42.9%) of 310 women were referred to and attended an HIV clinic within four months post-delivery and 95 (71.4%) of these had a CD4 cell count. Twenty (21.1%) had a CD4 cell count below 200 cells/mm^3^ and 13 (65.0%) were started on treatment sometime between diagnosis and four months post-partum. Because not all women attended all follow-up visits, the proportion reporting referral to and attendance at an HIV clinic and assessment with a CD4 cell count was examined at each visit (Table 1). The proportion receiving an HIV clinic referral post-delivery rose with time; 13.6% and 39.7% reported having received a referral after delivery when they were seen at the first and fourth post-natal visits, respectively. Of those who had been referred to an HIV clinic, the proportion who attended a clinic varied by follow-up visit, ranging from 45.9% at the one month post-partum visit to 68.0% at the three-month visit. The majority (64.9%−83.3%) of those attending an HIV clinic had had a CD4 cell count test performed, and about 20% had CD4 cell counts below 200 cells/mm^3^. Among these women eligible for HAART, only about half had initiated HAART at each visit.

**Figure 2:**
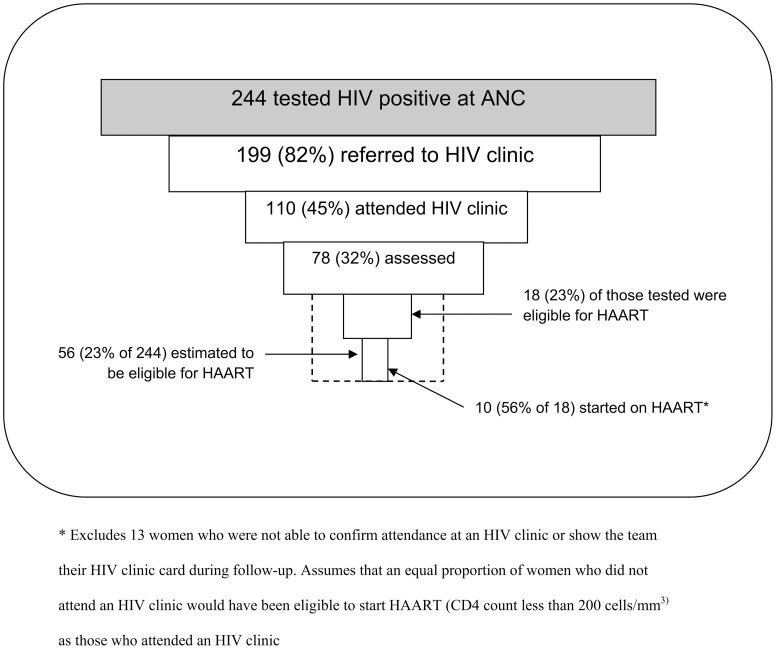
Attrition in the cascade of HIV assessment and treatment steps taken by women identified as HIV positive through PMTCT services.

Using data on the attrition along the patient cascade for HIV-positive pregnant women who attended the four month follow-up visit, the Piot-Fransen model (Figure 2) showed that 78 of 244 (32.0%) participants were referred, attended an HIV clinic and were assessed for their eligibility for HAART. Only 10 of 18 (55.5%) of those eligible for HAART had started it within four months of delivery.

**Table 1:** Proportion of HIV positive pregnant women receiving referral and attending an HIV clinic within 4 months post-partum.

		Received referral to HIV clinic			Attended an HIV clinic						
Follow up visit	Total seen	Pre-delivery	Post-delivery	Total (%)	Pre-delivery	Post-delivery	Total (% of those referred)	HIV clinic cards seen (%)^3^	CD4 test result (%)	CD4 count <200 cells/mm^3^	Started on HAART(%)
Enrolment	310^1^	123	0^2^	123 (51.3)	71	0^2^	71 (57.7)	36 (50.7)	30 (83.3)	5 (16.7%)	3 (60.0)
Post-partummonth 1	271 (87.4)	111	37	148 (54.6)	60	8	68 (45.9)	63 (92.6)	42 (66.7)	10 (23.8%)	5 (50.0)
Post-partummonth 2	245 (79.0)	103	48	151 (61.6)	68	23	91 (60.3)	74 (81.3)	48 (64.9)	8 (16.7%)	4 (50.0)
Post-partummonth 3	233 (75.2)	100	53	153 (65.7)	72	32	104 (68.0)	79 (76.0)	53 (67.1)	12 (22.6%)	7 (58.3)
Post-partummonth 4	244 (79.0)	102	97	199 (81.6)	74	49	123 (61.8)	98 (79.7)	78 (79.6)	18 (23.1%)	10 (55.6)

1 Comprises 240 women tested at the antenatal clinic and 70 women tested at the maternity ward around delivery. Excludes 93 women who were not diagnosed HIV positive for the first time through PMTCT screening in this pregnancy.

2 Referral post-delivery but pre-discharge from hospital.

3 Attended an HIV clinic and was issued with HIV clinic attendance/treatment card.

To estimate the potential total number in the entire study population who might have completed the cascade, based on data from the four month follow-up visit, we excluded 13 women who did not show their HIV clinic enrollment card during follow-up visits and therefore had no evidence of actually attending an HIV clinic. We assumed that the same proportion of women who did and did not attend an HIV clinic would have been eligible to start HAART (CD4 cell count less than 200 cells/mm^3^). Assuming 56 (23.1%) of 244 HIV-positive women were eligible to start HAART (Figure 2), then only 10 (17.6%) of HIV-positive women who were eligible for treatment actually navigated all the levels of the system and received ART within four months of delivery.

### Barriers and Factors Associated with Lack of Attendance at HIV Care and Treatment Services Pre-delivery

At enrollment, 52 (42.2%) of 123 women tested through PMTCT services who had received a referral to an HIV clinic prior to delivery had not attended prior to admission. Thirty-eight women were asked about their reasons for not attending an HIV clinic (others were not asked because they had not brought their HIV clinic cards to the maternity ward). Reasons included not understanding why it was important to attend an HIV clinic soon after diagnosis (n = 17), too ill to attend (n = 5), afraid to disclose their HIV status (n = 4), wanting to wait until after delivery (n = 4), distance to the HIV clinic (n = 2), being informed services were not available when they attended the HIV clinic (n = 2), lack of time (n = 1), did not believe the HIV results (n = 1), and not feeling it was necessary to attend HIV clinic services because she felt well (n = 1).

By four months post-partum, 199 women had been referred to an HIV clinic but 76 had not attended. On univariate analysis, there was strong evidence of an association of non-attendance was associated with being of non-Sukuma ethnicity, not having disclosed HIV status and poor compliance with PMTCT ART prophylaxis, moderate evidence of an association with having fewer antenatal visits, and weak evidence of an association with being Muslim and a primigravida (Table 2). On multivariate analysis (Table 3), lack of attendance at an HIV clinic was independently associated with failing to disclose HIV status (aOR = 3.06, 95%CI: 1.69–7.74), being of non-Sukuma ethnicity (aOR = 2.47, 95%CI: 1.28–4.76), and, to a lesser extent, with poor compliance with PMTCT ART prophylaxis (aOR = 2.13, 95% CI: 1.13–4.04), and having made fewer than 3 antenatal visits during pregnancy (aOR = 1.78, 95% CI: 0.93–3.38).

**Table 2:** Baseline factors at enrolment & univariate analysis of factors associated with not attending an HIV clinic four months post delivery.

Variable	Total at enrolment		Total seen at 4 months post-delivery		Not attended HIV clinicby 4 months post-delivery		Crude OR	95% CI	P
	N = 403	%	N = 199	%	n = 76	%			
**Age** 1									
<30 years	242	61.1	122	61.3	52	41.9	1		
> = 30 years	154	38.9	75	37.7	24	32.0	0.65	0.35–1.18	0.16
**Ethnicity**									
Sukuma	176	43.7	85	42.7	23	27.1	1		
Non-Sukuma	227	56.3	114	57.3	53	46.5	2.34	1.28–4.28	0.005
**Religion**									
Christian	304	75.4	152	76.4	53	34.9	1		
Muslim	97	24.1	47	23.6	23	48.9	1.34	0.96–1.86	0.08
No religion	2	0.5	0	-	0	-			
**Education level**									
No school	38	9.4	17	8.5	7	41.2	1		
Primary or higher	365	90.6	182	91.5	69	37.9	1.14	0.42–3.15	0.79
**Occupation**									
Unskilled/at home	388	96.3	188	94.5	72	38.3	1		
Skilled work	15	3.7	11	5.5	4	36.4	0.97	0.63–1.48	0.90
**Marital status**									
Married	285	70.7	147	73.9	57	38.8	1		
Not currently married	118	29.3	52	26.1	19	36.5	0.91	0.47–1.74	0.78
**Gravidity**									
Primigravidae	70	17.4	40	20.1	20	50.0	1		
Multigravidae	333	82.6	159	79.9	56	35.2	0.54	0.27–1.09	0.09
**HIV status disclosed**									
Yes	130^2^	36.5	157	78.9	51	32.5	1		
No	226^2^	63.5	42	21.1	25	59.5	3.06	1.51–6.16	0.001
**Partner tested for HIV**									
Yes	112	27.8	68	34.2	21	30.9	1		
No	291	72.2	131	65.8	55	42.0	1.69	0.87–3.01	0.13
**Number of ANC visits**									
3 or more visits	233^3^	39.9	124	62.3	40	32.2	1		
1 or 2 visits	155^3^	60.1	75	37.7	36	48.0	1.94	1.08–3.49	0.03
**Took PMTCT ART**									
Yes	260	27.0	127	63.8	38	29.9	1		
No	96	73.0	72	36.2	38	52.8	2.62	1.44–4.76	0.001
**Ever used contraception** 3									
Yes	208	51.6	93	46.7	39	41.9	1		
No	195	48.4	106	53.3	37	34.9	0.74	0.42–1.32	0.31
**Infant alive at 4 months**									
Yes	NA		179	89.9	67	37.4	1		
No			20	10.1	9	45.0	1.37	0.54–3.47	0.51

1 Excludes 7 women with unknown age.

2 Excludes 47 women who were tested on admission for delivery.

3 Excludes 15 women with no ANC card available at the maternity ward.

4 Oral and injectable hormonal contraception, condoms and intrauterine contraceptive device.

**Table 3:** Independent factors associated with not attending an HIV clinic four months post delivery^1^.

Variables	Adjusted OR^1^	95% CI	P
**Ethnicity**			
Sukuma	1		
Non-Sukuma	2.47	1.28–4.76	0.006
**Number of ANC visits during pregnancy**			
3 visits or more	1		
1 or 2 visits	1.78	0.93–3.38	0.08
**Took PMTCT tablets**			
Yes	1		
No	2.13	1.13–4.04	0.02
**Disclosure of HIV status**			
Yes	1		
No	3.06	1.69–7.74	0.001

1 Adjusted for age, tribe, total ANC visits during pregnancy, whether PMTCT medication was taken and disclosure of HIV status to any other person.

Challenges to implementation that were mentioned included a lack of tracking systems to record whether women were referred and attended an HIV clinic. Health workers reported that, because adult HIV care and treatment services were busy, pregnant women often were not enrolled and assessed for eligibility for HAART during pregnancy. One health worker reported that a particular HIV clinic also sometimes refused to enroll women who had been sent from the maternity ward to the HIV clinic in the same hospital during their admission for delivery. The HIV clinic staff mistakenly assumed that HIV-positive pregnant women had received all the care they required at the antenatal PMTCT services or in the maternity ward. There were few suggestions on how the system might be improved, but those mentioned included asking PMTCT health workers to accompany pregnant women to an HIV clinic, and designated HIV care and treatment services for pregnant women.

### Clinic Observations

In total, 134 pregnant women attended the nine observed antenatal HIV education sessions, facilitated by a nurse in Swahili. The average length and average number of participants for the sessions was 26 minutes (range 15 to 35 minutes) and 15 (range 7 to 22), respectively. No participants asked any questions in four of these sessions. Most questions in other sessions focused on PMTCT, and women were interested to learn about the effectiveness of ART for PMTCT. However, there were no questions about interventions that focused on the woman’s own health if she tested positive despite this not being mentioned in any of the sessions.

## Discussion

### Key Findings

Current WHO guidelines recommend that HIV-infected pregnant women should be initiated on HAART for their own health if their CD4 cell count is less than 350 cells/mm3 or if they are in WHO clinical stage 3 or 4 [23]. HIV testing is the key entry point to HIV care for both mother and child, and initiation of HAART, if required, should be done as soon as possible in pregnancy in order to give optimal health outcomes for both mother and child. It is clear from our study that, irrespective of the specific CD4 cell count threshold for initiating treatment, the majority of women testing HIV-positive at the main antenatal clinics in Mwanza who require HAART were unable navigate and complete the complex cascade of steps needed to ensure they start HIV treatment in pregnancy, even where PMTCT services and HIV treatment clinics were in the same town or even in the same hospital. At the pre-natal stage, attrition in the cascade was particularly severe between testing and receiving a referral to and initial attendance at an HIV clinic, being assessed for HAART eligibility and receiving HAART if eligible. The proportion receiving a referral improved with time since HIV testing for those staying in post-natal care but HIV clinic attendance, assessment and initiation of HAART continued to be problematic. If these findings are similar across other regions in the country, then with an HIV prevalence in antenatal attenders of 8.4% [18], the number of women who were not linked into long-term care in Tanzania will be substantial. Furthermore, the infants of many HAART-eligible women would not have received the most effective regimen for PMTCT if their mothers were significantly immuno-suppressed. In our setting fewer than 18% of pregnant women potentially eligible for HAART were assessed and initiated on HAART between HIV diagnosis in pregnancy and four months after delivery. Similar loss across the HIV diagnosis-treatment cascade has been observed in Kenya and other countries [24]–[27].

Concerns that the health of mothers has been neglected in PMTCT programmes have been raised previously, since programme emphasis had largely focused on preventing transmission to infants [28]. In our study, failures in the intervention steps were observed at several levels. Nearly half of all HIV-positive women who delivered in hospital had not been referred to an HIV clinic prior to delivery. None of the HIV-positive women tested at the maternity ward received a referral to an HIV clinic before discharge from the hospital, and many women, who had shown willingness to access health services by attending up to four post-natal RCH visits, were still not referred to or had attended an HIV clinic. Furthermore, women were rarely asked whether they had been referred to or had attended these services. Many did not bring their HIV clinic cards to the maternity suite or to their RCH clinic visits, making it difficult for maternity and RCH staff to confirm who had or had not been linked into the adult HIV treatment services.

### Individual-level Factors and Lack of Attendance at an HIV Clinic

Women who did receive referrals to an HIV clinic described a number of barriers to attending during pregnancy, such as lack of understanding why they should attend and fear of disclosure of their HIV status. The latter can be associated with stigma which has been shown to influence willingness to access HIV treatment services in other studies [29]–[31]. In addition to lack of disclosure, which has been associated with lack of attendance at services in some, but not all, studies [27], [31], both ethnic group and religion were associated with lack of attendance at a clinic. Ethnicity has been associated with health-seeking behaviours in previous studies in Tanzania [32], [33]. The Sukuma are the traditional residents of Mwanza Region. Women from other ethnic groups may have different economic or educational status or different cultural beliefs related to health care, HIV infection and its treatment, all of which might influence their uptake of HIV services. Muslim women were also less likely to attend an HIV clinic in the city than non-Muslim women, again perhaps because socio-cultural norms preclude them from seeking on-going care for this stigmatising disease [34].

Delays in accessing care after diagnosis amongst adult HIV patients are common in sub-Saharan Africa [35]–[37]. In one study of 701 Kenyan HIV-positive patients, 31.4% had delayed seeking care for over six months and 21.8% for over one year [38]. Being female, non-disclosure of HIV status and distances between the testing centres and the HIV care and treatment centres were associated with delayed attendance for HIV care. Only 35% of HIV-positive pregnant women in Kinshasa attended an HIV clinic within a median time of 4.5 months after HIV diagnosis [39]. The median time between HIV diagnosis and attendance was 2.5 months. Distance to the clinic, transportation costs, travel and non-disclosure were cited as the commonest reasons for missed visits. In our study, in addition to these four issues, infrequent antenatal clinic attendance was also strongly associated with lack of attendance for on-going care. Strategies to encourage disclosure, such as offering antenatal clinic couple counselling and testing services and improved support to women who are considering disclosure, should therefore be considered [40]–[42].

### Provider-level Factors and Lack of Attendance at an HIV Clinic

Lack of understanding among staff about when women should be referred after being given their HIV results is likely to have contributed to the low referral rate. Inadequate health worker knowledge was found to impact on a number of steps in the PMTCT care continuum in South Africa [43], and health workers in Malawi also sometimes failed to refer asymptomatic women to HIV clinics [44]. The majority of health workers in our study were aware of the HIV referral system but there were basic misunderstandings in how this should be implemented and it was unclear what post-test counselling messages had been delivered to mothers concerning maternal care. Content of HIV-related educational messages was also important. The Tanzanian national PMTCT guidelines on the information to be given during pre-test counselling focus on the benefits of HIV testing, MTCT, disclosure and the importance of assessing the mother for eligibility for HAART [20]. However this latter recommendation is not reinforced in antenatal HIV education sessions. Since women demonstrated a lack of understanding about the need to attend an HIV clinic for assessment soon after diagnosis, it is clear that strengthening antenatal and post-natal training and counselling will be essential to reinforce key messages about on-going health care for HIV-positive pregnant women. In addition, improvement of PMTCT services in the maternity suites will be essential to allow newly diagnosed HIV-positive women to receive information regarding their HIV-positive status and on-going care before discharge from the labour ward.

### Recommendations

There is evidence that HAART in pregnancy provides a substantially greater reduction in MTCT of HIV compared with simpler regimens. In South Africa, for example, MTCT of HIV was 11–13% with single-dose NVP compared to 0.3% in women who received more than 7 weeks of HAART [45], [46]. A combined analysis in South and West African cohorts of transmission through breastfeeding showed that there was a 5% cumulative risk of transmission to the infant even in women with high CD4 cell counts [47]. This would argue for countries to consider implementing the WHO Option B, where HAART may be started at ANC services irrespective of CD4 cell count, and continued until one week after breastfeeding has ended in women who are not eligible for lifelong HAART under current treatment guidelines [7]. This regimen allows the infant maximum protection from vertical transmission and ensures that women who do require HAART for their own health have an opportunity to start this as early as possible after diagnosis of their positive HIV status, without having to wait for their CD4 cell count test result [47], [48]. This is important since late treatment initiation in Africa has been associated with higher mortality [49], [50].

Even with WHO Option B, improvement in linkages between services will still be required to ensure that women are assessed to determine whether they need to stay on HAART after breastfeeding and to ensure they are linked into HIV care and support services to monitor their own health. Whilst our study demonstrated the importance of continuing to deliver information post-partum about enrollment into HIV treatment services, steps to link women into HIV care will have to happen more promptly and efficiently in order to avoid treatment interruptions. HAART may therefore need to be available at both ANC and RCH post-delivery services if these are not integrated. Strategies such as the *mothers2mothers* program which works with HIV-positive “mentor mothers” may also be effective in offering HIV-infected pregnant women additional support as they transition between ANC and adult HIV care and treatment services [51]–[53]. This could be especially valuable as women testing HIV-positive through antenatal services in Mwanza Region currently receive little or no psycho-social support. Decentralising HIV treatment services is underway in Tanzania and increasing availability of HIV care, especially in rural areas, and task-shifting to allow nurses to prescribe ART, may also improve linkage to care and more rapid initiation of treatment.

### Study Strengths and Limitations

Strengths of our study were the prospective cohort design and multiple data collection methods. Limitations include the fact that cohort participants were enrolled at delivery, and therefore the study did not include pregnant women testing HIV-positive in antenatal services but who did not deliver in hospitals. In addition, women from remote rural areas in Tanzania were not included and we were not able to determine if women lost in the treatment cascade sought care elsewhere. We have assumed that the proportion of HAART-eligible women who not attend an HIV clinic is the same as those who do attend a clinic but this assumption may be incorrect. The proportion of women who reported receiving a referral to an HIV clinic increased post-partum, and it is possible that the research team contributed to this improvement.

### Conclusion

In conclusion, we observed multiple missed opportunities to link HIV-positive pregnant women into HIV treatment programmes. Changes in the training and supervision of health workers implementing PMTCT and health system modifications are needed for staff to successfully enable women who test HIV-positive during antenatal or delivery care to access HIV treatment and care services for their own health, and to identify women who have not been assessed. Initiating HAART at the ANC for all pregnant women should reduce MTCT and maximise the health benefits for women who require HAART for their own care. Further research should be conducted to explore the effectiveness of these interventions and to explore the barriers to attending HIV care and treatment that pregnant women or mothers face when they leave PMTCT services, since linkage between the services will be essential at some point during pregnancy or the post-partum period.
